# Electrophysiologic and anti‐inflammatorial effects of cyclooxygenase inhibition in epileptiform activity

**DOI:** 10.14814/phy2.15800

**Published:** 2023-09-09

**Authors:** Canan Akunal Türel, Hümeyra Çelik, Ayhan Çetinkaya, İdris Türel

**Affiliations:** ^1^ Department of Neurology Bolu Abant Izzet Baysal University Medical School Bolu Merkez/Bolu Turkey; ^2^ Department of Physiology Alanya Alaaddin Keykubat University Medical School Antalya Turkey; ^3^ Department of Physiology Bolu Abant Izzet Baysal University Medical School Bolu Merkez/Bolu Turkey; ^4^ Department of Pharmacology Bolu Abant Izzet Baysal University Medical School Bolu Merkez/Bolu Turkey

**Keywords:** acute seizure, anti‐inflammatory, diclofenac potassium, electrophysiology, penicillin‐induced epileptiform activity

## Abstract

The aim of our study is to investigate the electrophysiological and anti‐inflammatory effects of diclofenac potassium on epileptiform activity, which is the liquid form of diclofenac, and frequently used clinically for inflammatory process by inhibiting cyclooxygenase enzyme (COX). Wistar rats aged 2–4 months were divided into Epilepsy, Diazepam, Diclofenac potassium, and Diazepam+diclofenac potassium groups. Diazepam and diclofenac potassium were administered intraperitoneally 30 min after the epileptiform activity was created with penicillin injected intracortically under anesthesia. After the electrophysiological recording was taken in the cortex for 125 min, interleukin‐1β (IL‐1β), interleukin‐6 (IL‐6), and tumor necrosis factor‐α (TNF‐α) were evaluated by the ELISA in the serums. No change was observed between the groups in serum IL‐1β, IL‐6, and TNF‐α values. It was observed that the co‐administration of diclofenac potassium and diazepam at 51–55, 56–60, 61–65, 111–115, and 116–120 min was more effective in reducing spike amplitude than diclofenac potassium alone (*p* < 0.05). Single‐dose diclofenac potassium did not have an anti‐inflammatory effect in epileptiform activity but both diazepam and diclofenac potassium reduced the epileptiform activity.

## Graphical abstract

We evaluated the electrophysiological and anti‐inflammatory effects of diclofenac potassium, the liquid form of diclofenac, based on the hypothesis that epileptic seizure is inflammatory and therefore anti‐inflammatories can be good for epileptic seizures. Although diclofenac potassium with diazepam decreased the spike frequency and spike amplitudes of the seizure electrophysiologically, it could not decrease the serum inflammation markers.

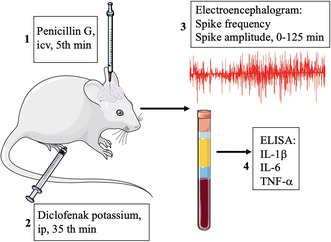

## 1 INTRODUCTION

Epilepsy is a serious neurological, psychological, and cognitive disease caused by the spontaneous firing of hyperexcitable neurons, resulting in recurrent seizures (Bambal et al., 2011; Stefanescu et al., 2012). Although many environmental and genetic factors have been suggested, the exact pathophysiology for the occurrence of epilepsy is still unclear (Shorvon, 2014). Currently, the first‐line treatment of epilepsy is symptomatic, aimed at reducing neuronal excitability by inhibiting sodium ion channels or influx of chloride ions, which increases the activity of aminobutyric acid (GABA) receptors (Goldenberg, 2010). For this purpose, benzodiazepines are the most widely used drugs as they have anticonvulsant activity at epilepsy. Diazepam (DZP), a common medicine from the benzodiazepine class with a prolonged action, is frequently chosen for patients who may need long‐term care (Huemer et al., 2010; Strac et al., 2008). However, in the treatment of epilepsy, although 30% of patients are resistant to these treatments, antiseizure drugs have many side effects (Stafstrom & Carmant, 2015).

Traumatic brain injuries, cerebrovascular accidents, infections of the central nervous system, and strokes are among the neurological insults that cause up to 60% of cases of epilepsy, where inflammation is a crucial component of epileptogenesis (Klein et al., 2018). Increasing clinical and experimental evidence indicates that inflammatory processes in the brain play an important role in epileptic seizures (Riazi et al., 2010). It is possible for inflammatory processes to start in the central nervous system‐local or to spread from systemic circulation due to a blood–brain barrier breach (Choi & Koh, 2008). The expression and activation of numerous inflammation‐related enzymes, such as inducible nitric oxide synthase, NADPH oxidase, cyclooxygenase, caspases, and matrix metalloproteinases, as well as the subsequent release of cytokines (such as IL‐1, IL‐6, and TNF‐α), prostaglandins, and chemokines, can all be triggered by inflammatory triggers (e.g., the NF‐κB pathway) (Green et al., 1994; Iñiguez et al., 1999). When microglia are activated, COX, one of the enzymes, produces inflammatory mediators by biosynthesizing prostaglandins from arachidonic acid (Akundi et al., 2005). Cyclooxygenase consists of the enzymes COX‐1 and COX‐2 that are regarded as proinflammatory and leads to the production of inflammatory cytokines (IL‐1, IL‐6, and TNF‐α) (Linton & Fazio, 2008).

The knowledge that neuroinflammation plays a role in the pathogenesis of epilepsy has formed the basis for the use of steroids and other anti‐inflammatory treatments for anticonvulsant purposes in drug‐resistant epilepsy (Wheless et al., 2007). Diclofenac is a nonsteroidal anti‐inflammatory (NSAID) drug with a low molecular weight (Altman et al., 2015). By inhibiting COX enzymes 1 and 2, it inhibits the release of arachidonic acid and thus the release of prostaglandins that play a role in inflammation (Smyth et al., 2009). Diclofenac potassium is a fast‐acting agent available in liquid form as a derivative of diclofenac (Moore et al., 2015). In clinical practice, it is widely used as an analgesic and antipyretic, to relieve postoperative pain (Ghlichloo & Gerriets, 2021). The effectiveness of nonsteroidal anti‐inflammatories in neuroinflammation is known (Fielder et al., 2020). Trying to manage the epileptic seizure with the liquid form of diclofenac potassium, which we have chosen to meet the need for anticonvulsant drugs for new targets with different mechanisms of action‐by modulating the proinflammatory cytokine response may create a new strategy in the treatment of epilepsy. For this purpose, the electrophysiological, anti‐inflammatory, and anticonvulsant effects of diclofenac potassium on the epileptiform activity experimentally induced with penicillin in rats will be investigated in our study.

## 2 METHODS

The ethics committee approval of the study (decision number: 25/2020) was obtained from Bolu Abant İzzet Baysal University (BAIBU) Experimental Animals Local Ethics Committee. In the study, Wistar‐Albino breed 2‐month‐old 200–250 g male rats were obtained from BAIBU Experimental Animals Center and maintained at a temperature of 19 ± 2°C and relative humidity of 50–70 with ad libitum water and pallet food in a 24‐h light/24‐h dark cycle. Rats were caged in groups, and four groups were formed with *n* = 8. The groups in the study were Epilepsy (Control group, formed with penicillin), Diazepam (Epilepsy + Diazepam, positive control), Diclofenac Potassium (Epilepsy + Diclofenac Potassium, drug), Diclofenac Potassium + Diapezam (Epilepsy + Diclofenac Potassium + Diazepam, synergy). All experiments were carried out between 08:00 and 12:00.

### 2.1 Epileptiform activity induction and drug applications

To create an epileptiform activity with penicillin, animals that were fasted 24 h ago were fixed on the operating table after they were shaved from the top of their heads under urethane anesthesia. The scalp of the rats was opened in the rostro‐caudal direction, approximately 3 cm in length, with a scalpel. The soft tissue under the left cortex scalp was removed by electrocautery. The skull bone was removed by making circular movements with a touring motor and thinning it. After the electrodes were placed and basal activity was recorded for 5 min, 500.000 IU penicillin (2.5 μL, icv) (Aygun et al., 2020) was administered intracortically to the somatomotor cortex with a Hamilton injector (701 N, Hamilton Co.) to induce epileptic activity. The injection coordinates were 2 mm lateral, 1 mm anterior, and 1.2 mm depth of the bregma line. To the epilepsy group, sham saline 0.9%, 0.1 mL, i.p. was given. After 30 min, diazepam (0.1 mL, 5 mg/kg, i.p.) (Vito et al., 2014) and diclofenac potassium (10 mg/kg i.p.) (Elgarhi et al., 2020) were administered to the Diclofenac potassium, Diazepam, and Diclofenac potassium + Diazepam groups.

### 2.2 Electrophysiological assessment

For electrophysiological recording, two Ag/AgCl ball electrodes (one positive) were placed 1 mm anterior to the bregma and 2 mm lateral to the sagittal suture. The negative one was placed 5 mm posterior to the bregma and 2 mm lateral to the sagittal suture. For grounding, 1 Ag/AgCl clamp electrode was fixed to the right auricle by applying recording gel. The activity taken with the electrodes was amplified in the BioAmp (ADInstruments) interface and instantly transferred to the PowerLab 4/SP (ADInstruments) data acquisition unit and recorded. After the analog signals were converted to digital, they were transferred to the computer and analyzed. The recording was taken for 120 min after the seizure was created. We showed the spike frequency by counting number of burst and the spike amplitudes by calculating the amplitude in mV from the obtained electrophysiological activity record.

### 2.3 Blood collection and ELISA

**FIGURE 1:**
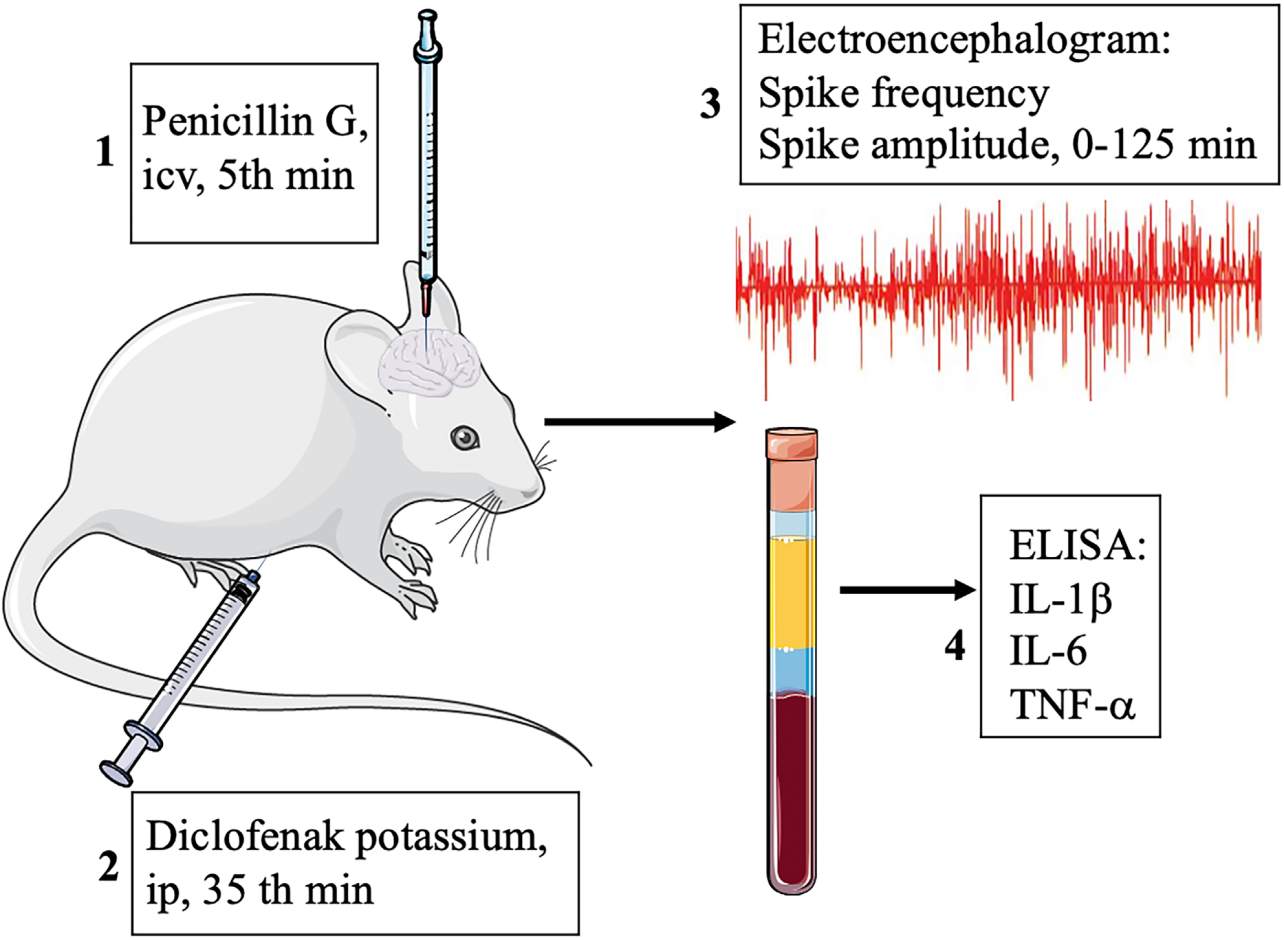
Experimental procedure.

After electrophysiological recordings were made, 5 mL of blood was taken intracardiacly from all rats. After the blood in the tube was centrifuged at 1700 *g* for 10 min, it was stored in Eppendorf tubes at –80°C until the biochemical parameters were studied. Serum levels of IL‐1β, IL‐6, and TNF‐α were determined by using commercially available enzyme‐linked immunosorbent assay (ELISA) kits (BT LAB Bioassay Technology Laboratory) according to the manufacturer's instructions (Figure 1).

### 2.4 Statistical analysis

Data were analyzed in the statistical package program IBM SPSS Statistics 25.0 (IBM Corp.). Descriptive statistics were given as the number of units (*n*), percent (%), mean ± standard deviation (*x* ± SD), and median (Q1–Q3) values. The normal distribution of the data of numerical variables was evaluated with the Shapiro–Wilk test. Comparisons between groups were made with one‐way analysis of variance (ANOVA) for normally distributed variables, and Kruskal–Wallis analysis for non‐normally distributed variables. Tukey HSD was used for normally distributed variables and Bonferroni corrected Mann–Whitney *U*‐test was used for non‐normally distributed variables as a multiple comparison test. A *p*‐value of <0.05 was considered statistically significant.

## 3 RESULTS

**FIGURE 2:**
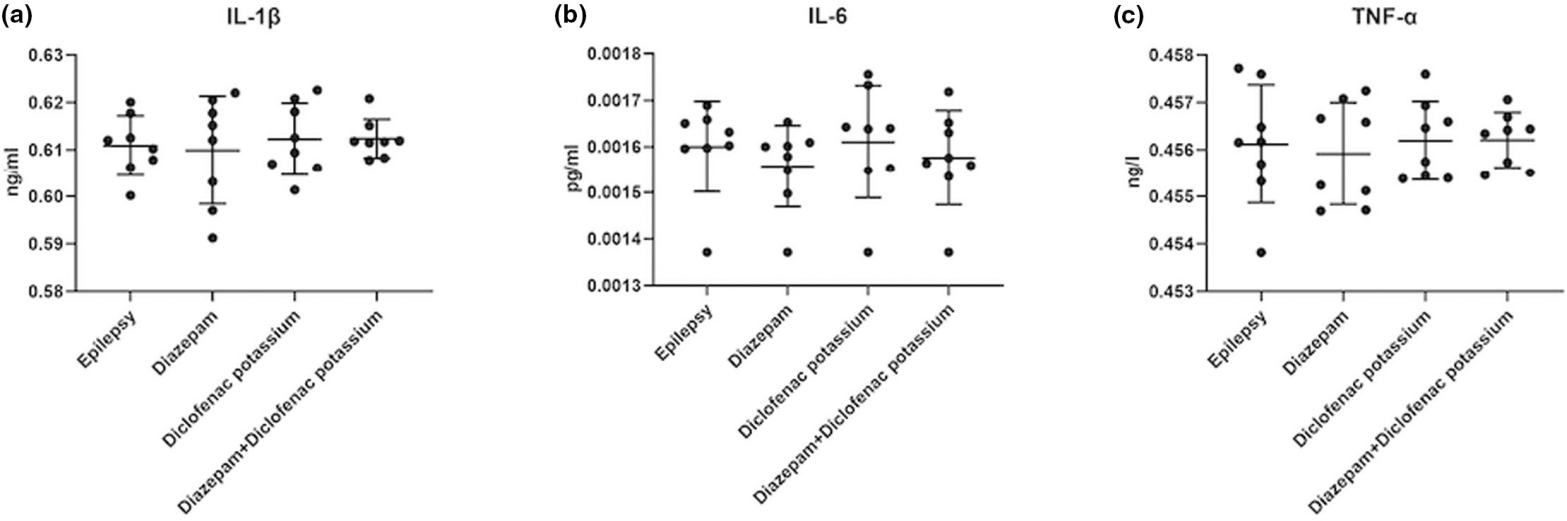
Comparison of proinflammatory cytokines IL‐6 (a), IL‐1β (b), and TNF‐α (c) means between groups (*n* = 8 per group).

In the study, in which the effects of diclofenac potassium on epileptiform activity were evaluated, no statistically significant change was found in IL‐1β, IL‐6, and TNF‐α values between the groups in inflammatory markers studied by ELISA in serum samples (*p* > 0.050) (Figure 2).

The electrophysiological results were evaluated; the mean spike frequency measured in the first 5 min of epileptic activity was similarly not statistically significant in all groups (*p* > 0.050) and like our other research (Akunal Türel et al., 2022; Danis et al., 2023). The mean values of spike frequency and spike amplitude recorded at 5‐min intervals from 30th minute to 125th minute are given in Table 1.

**TABLE 1:** The mean and median values of spike–wave and spike amplitude recorded at 5‐min intervals from 30th minute to 125th minute‐after the epileptiform activity.

Time (minute)	Epilepsy (*n* = 8)		Diazepam (*n* = 8)		Diclofenac potassium (*n* = 8)		Diazepam+diclofenac potassium (*n* = 7)		*p*‐value	
	Spike frequency	Amplitude (mV)	Spike frequency	Amplitude (mV)	Spike frequency	Amplitude (mV)	Spike frequency	Amplitude (mV)	Spike frequency	Amplitude (mV)
0–5	0 ± 0	0.7 ± 0.5	0 ± 0	0.6 ± 0.2	0 ± 0	0.7 ± 0.2	0 ± 0	0.5 ± 0.2	0.00	0.78
6–10	36.3 ± 31.5	1.7 (0.3–2.1)	25.8 ± 18.7	0.63 (0.4–2.5)	38.1 ± 26.8	1.3 (0.2–2.8)	12 ± 8.1	1.1 (0.3‐1.8)	0.48	0.50
11–15	149.8 ± 62.1	2.6 ± 1.3	138.3 ± 61.8	2.4 ± 1.8	181.0 ± 90.0	2.3 ± 1.2	126.0 ± 69.2	2.1 ± 1.2	0.92	0.94
16–20	241.0 ± 89.9	3.1 ± 1.6	246.1 ± 96.1	3.1 ± 1.8	225.5 ± 69.9	3.0 ± 1.4	229.1 ± 66.1	2.8 ± 1.6	0.08	0.97
21–25	236.0 (98.0–351.0)	4.1 (1.1‐5.2)	321.5 (73.0–371.0)	3.0 (1.2–5.6)	220.0 (90.0–264.0)	3.3 (1.0–6.1)	189.0 (140.0–360,0)	3.0 (0.7‐5.3)	0.07	0.94
26–30	232.0 (93.0–394.0)	3.6 ± 1.7	33.5 (69.0–395.0)	3.1 ± 1.5	177.5 (116.0–269.0)	3.1 ± 1.7	235.0 (154.0–315.0)	3.3 ± 2.0	0.06	0.94
30–35	202.5 ± 63.4	3.5 ± 4.1	294.6 ± 119.8	3.2 ± 1.5	190.3 ± 65.8	3.1 ± 1.5	267.5 ± 107.4	2.7 ± 1.8	0.13	0.55
36–40	185.3 ± 69.3	3.8 (1.2–5.3)	238.5 ± 92.1	2.7 (0.8–4.9)	168.3 ± 59.6	3.0 (1.0–5.3)	221.2 ± 50.0	2.3 (0.8–4.0)	0.15	0.47
41–45	190.5 (109.0–285.0)	3.3 ± 1.4	178.0 (51.0–276.0)	2.7 ± 1.4	149.5 (14.0–240.0)	2.8 ± 1.08	173.0 (111.0–377.0)	2.6 ± 2.0	0.88	0.12
46–50	175.5 ± 63.9	3.3 ± 1.3	144.5 ± 64.8	2.5 ± 1.3	152.2 ± 46.7	2.7 ± 0.9	156.8 ± 124.1	2.5 ± 2.0	0.67	0.09
51–55	183.5 ± 60.7	3.2 ± 1.2	147.0 ± 60.0	2.3 ± 1.3	155.0 ± 40.9	2.7 ± 0.8	144.8 ± 128.6	2.2 ± 1.8	0.57	0.04
56–60	182.1 ± 52.3	3.4 ± 1.3	118.3 ± 56.3	2.0 ± 1.2	143.3 ± 33.2	2.5 ± 0.7	135 ± 120.9	2.2 ± 1.8	0.21	0.03
61–65	204.7 ± 78.2	3.1 ± 1.2	113.6 ± 55.1	1.9 ± 1.3	139.7 ± 38.0	2.2 ± 0.7	126.0 ± 112.1	2.2 ± 1.8	0.12	0.02
66–70	222.3 ± 117.3	2.8 ± 1.2	102.0 ± 55.9	1.9 ± 1.2	128.3 ± 39.8	2.3 ± 0.8	120.2 ± 110.4	2.1 ± 1.8	0.07	0.06
71–75	209.1 ± 107.7	2.8 ± 1.2	98.2 ± 59.9	1.7 ± 1.1	129.1 ± 41.7	2.0 ± 0.7	112.7 ± 115.1	2.0 ± 1.7	0.09	0.05
76–80	203.5 ± 93.1	2.9 ± 1.2	90.3 ± 65.6	1.6 ± 1.0	127.7 ± 44.6	2.0 ± 0.8	105.8 ± 111.9	1.9 ± 1.7	0.08	0.06
81–85	184.8 ± 79.9	4.1 ± 1.9	74.7 ± 55.9	1.5 ± 1.0	128.5 ± 43.8	1.9 ± 0.7	106.7 ± 82.3	2.0 ± 1.6	0.06	0.05
86–90	195.1 ± 99.1	2.0 (1.6–4.1)	67.3 ± 55.8	1.0 (0.3–3.3)	126.0 ± 52.4	1.7 (0.9‐3.3)	102.0 ± 90.1	1.4 (0.3–3.6)	0.05	0.06
91–95	164.5 (78.0–330.0)	1.6 (1.3–3.9)	25.0 (6.0–189.0)	1.1 (0.2–3.3)	122.0 (67.0–243.0)	1.7 (0.7–4.1)	88.0 (1.0–273.0)	1.4 (0.3–3.5)	0.03	0.08
96–100	168.0 (42.0–334.0)	1.8 (1.4–4.6)	22.0 (1.0–177.0)	0.9 (0.3–3.0)	143.0 (51.0–192.0)	1.5 (0.7–3.1)	49.0 (1.0–258.0)	1.4 (0.2–3.4)	0.03	0.09
101–105	180.5 (22.0–296.0)	2.4 ± 1.1	16.0 (4.0–159.0)	1.0 ± 0.9	131.0 (29.0–174.0)	1.7 ± 1.5	49.0 (0.0–226.0)	1.5 ± 1.2	0.02	0.08
106–110	177.2 ± 92.4	1.9 (0.5–4.4)	42.6 ± 49.5	0.7 (0.2–3.0)	96.2 ± 73.3	1.3 (0.7–2.8)	88.8 ± 65.3	1.1 (0.0–3.0)	0.04	0.05
111–115	191.5 (4.0–270.0)	1.5 (0.4–4.6)	7.5 (0.0–144.0)	0.72 (0.2–2.7)	84.5 (0.0–184.0)	1.4 (0.7–3.1)	48.0 (0.0–228.0)	0.9 (0.0–3.2)	0.06	0.03
116–120	175.0 (0.0–285.0)	2.0 ± 1.3	8.5 (0.0–113.0)	0.9 ± 0.7	90.0 (0.0–176.0)	1.4 ± 0.7	36.0 (2.0‐233.0)	1.2 ± 1.0	0.04	0.02
121–125	129.0 (0.0–296.0)	1.5 (0.8–4.4)	9.0 (0.0–96.0)	0.5 (0.0–1.7)	84.0 (0.0–143.0)	1.0 (0.5–2.6)	43.0 (0.0–156.0)	0.8 (0.4–2.6)	0.05	0.01

**FIGURE 3:**
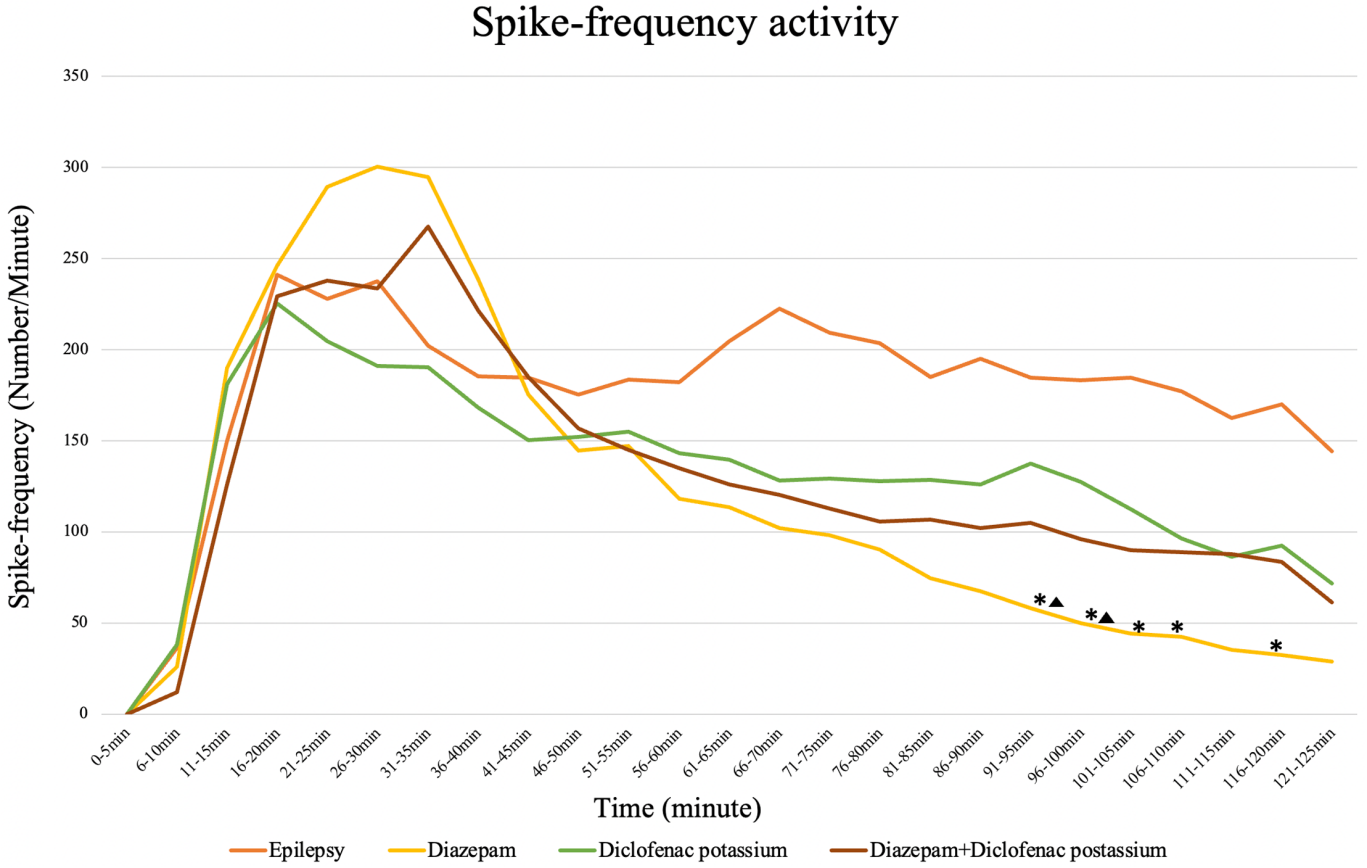
Measurement of spike frequencies among groups during 125 min *Diazepam is more effective than diclofenac potassium, *p* < 0.050. ▲ The diazepam group have less spike frequency than the epilepsy group (*p* < 0.050).

In the recording, a statistically significant decrease was observed in the median values of spike frequency of diazepam compared to the control between 91–95 (*p* = 0.006), 96–100 (*p* = 0.005), 101–105 (*p* = 0.003), 106–110 (*p* = 0.005), and 116–120th (*p* = 0.004) minutes. It is important that the data show the accuracy of our model. In addition, diazepam alone was statistically more effective than diclofenac potassium in spike frequency at 91–95th (*p* = 0.048) and 96–100th (*p* = 0.049) minutes (Figure 3).

**FIGURE 4:**
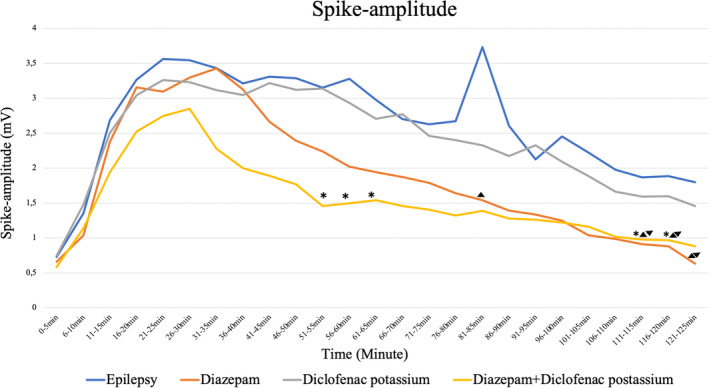
Spike amplitude graph between groups during 125 min of measurement. ▲ Diazepam statistically significant decreased spike‐amplitude values compared to the control, (*p* < 0.050). ▼ Diazepam was found to be more effective in reducing spike‐amplitude values than diclofenac potassium, (*p* < 0.050). *The co‐administration of diclofenac potassium and diazepam resulted in a statistically significant decrease compared to diclofenac potassium alone (*p* < 0.050).

**FIGURE 5:**
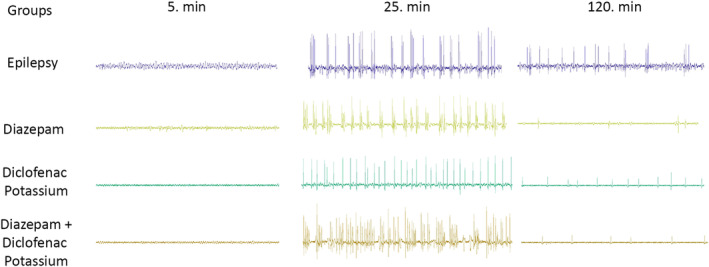
Electrical recording traces.

It was determined that the co‐administration of diclofenac potassium and diazepam at 51–55 (*p* = 0.015), 56–60 (*p* = 0.028), 61–65 (*p* = 0.037), 111–115 (*p* = 0.022), and 116–120th (*p* = 0.039) minutes of EEG measurements resulted in a statistically significant decrease in spike‐amplitude values when compared to diclofenac potassium alone. As a finding that supports the accuracy of our model, diazepam statistically significant decreased spike‐amplitude values compared to the control at 111–115 (*p* = 0.045), 116–120 (*p* = 0.045), and 121–125th (*p* = 0.008) minutes. Diazepam was found to be more effective in reducing spike‐amplitude values than diclofenac potassium at 111–115 (*p* = 0.021), 116–120 (*p* = 0.028), and 121–125th (*p* = 0.021) minutes (Figures 4, 5).

## 4 DISCUSSION

According to our study results, a single dose of diclofenac potassium in the epileptiform activity induced with penicillin does not show an anti‐inflammatory effect in serum, and IL‐1β, IL‐6, and TNF‐α values did not decrease. In the electrophysiological examination, diclofenac potassium was not more effective than diazepam, but when diazepam and diclofenac potassium were used together, more anticonvulsant results were observed in reducing spike amplitude compared to diclofenac potassium and diazepam alone.

Experimental and clinical studies have proven that neuroinflammation plays a major role in epilepsy (Maroso et al., 2011). These are a connection between inflammation and seizures, and indicate that inflammation is not just an epiphenomenon of epilepsy but may actively contribute to the pathology (Vezzani et al., 2012). It is known that inflammatory mediators lower the seizure threshold by increasing the expression of genes related to neuronal cell death and synaptic plasticity (Vezzani et al., 2013), and cytokine levels are high in the cerebrospinal fluids of epileptic rats (Gomez et al., 2004). The COX‐1 and COX‐2 isoforms of the enzyme COX, which limits prostaglandin synthesis, are low in the normal brain but increase after damage due to inflammation (Choi et al., 2009). The anti‐inflammatory effect of diclofenac potassium by inhibiting the COX‐1 and COX‐2 enzyme can be considered as a good solution for neuronal excitability caused by inflammation in epilepsy. Thus, cytokine production (IL‐6, IL‐1β, and TNF‐α) in inflammatory pathways triggered by the COX enzyme can be suppressed.

In a study, diclofenac decreased IL‐1β and TNF‐α values in brain homogenates in the kindling epilepsy model created with pentylenetetrazol (Elgarhi et al., 2020). As the first study evaluating the effect of diclofenac potassium on penicillin‐induced epileptiform activity, a single dose of diclofenac potassium could not produce the expected anti‐inflammatory effect in serum. At this stage, in a situation originating from the central nervous system such as epilepsy, it seems more targeted to choose the central tissue to evaluate the efficacy of the peripherally administered drug. Because even if statistical significance was not detected, contrary to expectations, diclofenac potassium given after seizures increased in serum IL‐1β, IL‐6, and TNF‐α values in graphs. Interestingly, in the study of Vieira et al., in the kindling epilepsy model created with pentylenetetrazole, a GABA‐A agonist is a dose‐dependent molecule used to induce chronic, subacute, and acute experimental epileptic seizures, systemic convulsant (Shimada & Yamagata, 2018). Similarly, diclofenac at the same dose caused the same results as ours in serum, despite 15 days of chronic administration (Vieira et al., 2016). They approached the unexpected increase in serum of diclofenac, which causes a decrease in IL‐1β and TNF‐α in the cortex and hippocampus tissues, as a compensatory increase in the increased IL‐10 in seizures (Youn et al., 2012). If we interpret this increase by evaluating the different response system of the immune system and the current experimental environment, penicillin applied for seizure formation was given intracerebrovascularly and diclofenac was given intraperitoneally. Every trauma (Brøchner & Toft, 2009) and any chemical molecule given to the body (Zhang & An, 2007) cause an increase in proinflammatory cytokines in the acute period. From another point of view, kidney damage by anti‐inflammatory drugs‐diclofenac (Peter & Evan Prince, 2018) may explain the increased inflammation in the serum.

Penicillin‐induced experimental epileptogenic activity is an acute model that allows EEG recording by applying penicillin to the cortical surface‐ mimics grand mal epilepsy. It starts locally and turns into generalized seizure in a synchronous manner (Fisher, 1989; Sagratella et al., 1985). Penicillin inhibits the synaptic transmission of GABA by binding to GABA_A_ receptors to which GABA binds. GABA_A_ receptors are ligand‐gated chloride channels, inhibiting the flow of chloride into the cell is the widely accepted mechanism (Barrons et al., 1992; Chow et al., 2005; Sutter et al., 2015). Penicillin is a local convulsant that stands out compared to others for investigating the cellular basis of spike frequency and the role of cortex and subcortical structures in spike frequency generation (Bambal et al., 2011; Marangoz, 1997). It has been reported that the effects of diclofenac potassium on epileptic seizures vary as proconvulsant and anticonvulsant depending on the selected form of diclofenac, the dose (Suemaru et al., 2018), the type of experimental model (Akarsu et al., 2006), and protocol (Dhir et al., 2006). In the maximal electroshock‐induced seizure model, 20 mg/kg diclofenac given ip 30 min before the experiment increased the seizure score and frequency more than the 10 mg/kg dose (Suemaru et al., 2018). In addition, aceclofenac given as a diclofenac derivative for penicillin‐induced epileptiform activity increased the spike frequency values by showing a proconvulsant effect in electrophysiological examination (Taşkıran et al., 2017). The convulsive threshold reduction of 10 mg/kg diclofenac given orally 2 hours before PTZ injection (Steinhauer & Hertting, 1981) is an example of the proconvulsive effects of diclofenac. In Theiler's murine encephalomyelitis virus epilepsy model, diclofenac injection at 5–10 mg/kg doses twice a day for 4 days did not affect the frequency and severity of seizures (Metcalf et al., 2021). In another study, diclofenac given by ip 30 min before PTZ injection in a PTZ kindling epilepsy model significantly decreased the seizure score and duration and increased latency (Elgarhi et al., 2020). Both 5 and 10 mg/kg doses of diclofenac sodium have been shown to reduce the complexity of PTZ‐induced epileptic seizures, the absence of a statistical difference between diazepam and diclofenac potassium was interpreted as having similar effects on seizures (Vieira et al., 2016). In our study, however, a time period in which diclofenac potassium was superior to diazepam or control was not observed, although there were minutes when there was no statistical difference in spike frequency and amplitude frequency between diazepam and diclofenac potassium. The graphs show that diclofenac potassium was not superior or equal to diazepam. In addition, it was a surprising finding that the combined use of diazepam and diclofenac potassium was more effective as an anticonvulsant than diazepam in our study. The occurrence of this finding 10–15 min after diclofenac potassium was given corresponds to the interval when diclofenac potassium begins to act (Reiner et al., 2001). Similar to our results, it is known that the use of valproic acid and diclofenac is more effective than valproic acid alone (Elgarhi et al., 2020). In the literature, it has been shown that diclofenac opens KCNQ2/3 potassium channels (Peretz et al., 2005) and potentiates retigabine in KCNQ2/3 opening (Khattab et al., 2018). In this respect, it is more appropriate to interpret diclofenac potassium as showing its rapid effects through voltage‐gated channels. Also, acute use of selective COX‐2 inhibitors potentiates the anticonvulsant effect of diazepam in relation to the pharmacodynamic type (Almaghour et al., 2014). In another respect, the combination of anticonvulsant and anti‐inflammatory effect may explain reducing spike amplitude.

The shortage of electrophysiological evaluation time, absence of central tissue for anti‐inflammatory response, and lack of ion channel screening like patch clamp were our limitations to comprehend the specific effects of diclofenac K in the amplitude changes of EEG. Our advantage is that this is the first study to evaluate the effects of diclofenac potassium in epileptiform activity in a time‐dependent and to give spike frequency and amplitudes.

Future experimental and clinical studies are needed in line with our recommendations to recommend diclofenac potassium both as an anticonvulsant and as a safe analgesic anti‐inflammatory drug.

## AUTHOR CONTRIBUTIONS

Investigation: Canan Türel, Ayhan Çetinkaya, İdris Türel; Method: Ayhan Çetinkaya, İdris Türel, Canan Türel; Analyze: İdris Türel, Hümeyra Çelik, İdris Türel; Writing: Canan Türel, Hümeyra Çelik, İdris Türel; Supervisor: Ayhan Çetinkaya; Critical Review: İdris Türel.

## ETHICS STATEMENT

The ethics committee approval of the study (decision number: 25/2020) was obtained from Bolu Abant Izzet Baysal University (BAIBU) Experimental Animals Local Ethics Committee.

## FUNDING INFORMATION

This study was supported by Bolu Abant Izzet Baysal University with the grant number 2021.08.32.1488.

## CONFLICT OF INTEREST STATEMENT

The authors declare no conflicts of interest.

## Data Availability

All data are availability if requested.
